# Cone-beam CT-derived parenchymal blood volume imaging in transarterial chemoembolization for hypovascular hepatic metastases: a case report

**DOI:** 10.3389/fonc.2026.1778300

**Published:** 2026-03-10

**Authors:** Hao Wang, Xiaotong Liu, Ying Liu, Encheng Liu, Bing Lv, Yilong Jiao, Xunjin Zeng, Bin Gao, Haonan Zhang, Haijun Gao, Guang Chen

**Affiliations:** 1 Department of Interventional Radiology, Tianjin First Center Hospital, Tianjin, China; 2 Tianjin Institute of Imaging Medicine, Tianjin, China; 3 Tianjin Organ Transplant Research Center, Tianjin, China

**Keywords:** cryoablation, embolization guidance technology, hypovascular liver metastases, PBV imaging, TACE

## Abstract

This case report describes the innovative integration of Cone-beam CT (CBCT) – derived parenchymal blood volume (PBV) mapping with embolization guidance technology to successfully direct the combined treatment of transarterial chemoembolization (TACE) and cryoablation of in a 70-year-old female with recurrent, hypovascular gallbladder liver metastases. By utilizing PBV software to quantitatively analyze tumor perfusion patterns through cone-beam CT acquisitions, the interventional team overcame the diagnostic limitations of conventional angiography in visualizing these typically occult lesions, identifying a characteristic peripheral hyperperfusion signature. Subsequently, the embolization guidance software automatically detected the tumor-feeding arteries and projected this information onto real-time fluoroscopy, enabling precise superselective catheterization. Post-TACE lipiodol deposition served as a radiopaque marker for accurate cryoablation probe placement under CT guidance. The procedural endpoint was objectively confirmed by a quantifiable change in PBV values, transitioning from pre-operative hypoperfusion to a post-operative “black cavity” pattern, indicative of complete treatment response. This novel multi-modal image-guided approach establishes a new paradigm for managing complex hepatic metastases, providing a comprehensive single-session solution for lesion identification, targeted embolization, and verified ablation.

## 1 Introduction

Gallbladder cancer is the most common malignant tumor of the biliary tract, characterized by its high malignancy and frequent late-stage diagnosis (1). At the time of diagnosis, approximately 60% of patients present with local invasion or distant metastases, with hepatic involvement being the most common site (2). Surgical resection remains the only potentially curative option for gallbladder cancer with liver metastases (1, 3, 4). For patients with postoperative recurrence, locoregional therapy serves as a crucial component of the comprehensive treatment strategy (5).

Transcatheter arterial chemoembolization (TACE), which delivers high-dose chemotherapeutic agents directly into the tumor-feeding arteries while minimizing systemic adverse effects, is widely used to control intrahepatic metastatic progression (6). Cryoablation, which induces physical tumor destruction through freeze-thaw cycles, can be combined with TACE to enhance local control (7, 8). Such a combination of locoregional therapies may yield synergistic effects, providing more effective treatment options.

However, the hypovascular nature of metastatic liver lesions and their feeding arteries make it difficult to visualize on conventional two-dimensional digital subtraction angiography (DSA), which poses a major obstacle to targeted therapy. Parenchymal blood volume (PBV) is a quantitative perfusion parameter derived from CBCT on DSA systems. While it correlates strongly with CT perfusion in hepatocellular carcinoma (HCC) and predicts mid-term outcomes after TACE, its application for locating hypovascular lesion and identifying feeder vessels- especially when combined with embolization guidance software - remains underexplored.

In this case report, we utilized a combined approach of PBV and embolization guidance software during TACE procedures. This integration provided real-time procedural assistance by enabling clear lesion identification through comparison of contrast-enhanced CBCT with PBV perfusion maps. Furthermore, the embolization guidance software automatically delineated the feeding arteries and superimposed this vascular roadmap onto live fluoroscopy for enhanced navigation.

## 2 Case report

A 70-year-old female was admitted following the discovery of a liver space-occupying lesion during a routine physical examination. Contrast-enhanced CT revealed masses in hepatic segments IV and V, suspected to be malignant with gallbladder involvement. One week later, the patient underwent resection of liver segment V and segment VIB, along with peripheral lymph node dissection. Postoperative pathology confirmed primary gallbladder carcinoma with hepatic invasion. Over the subsequent year, the patient received multiple cycles of combined immunotherapy and targeted therapy (camrelizumab plus capecitabine), as well as capecitabine monotherapy. One-year- follow-up abdominal contrast-enhanced CT revealed a new nodule adjacent to the residual liver resection margin, suggestive of recurrent metastasis. The patient’s liver function was classified as Child-Pugh Class A with a normal coagulation profile. Based on a comprehensive evaluation, the multidisciplinary team opted for a local therapeutic approach integrating TACE with ablation.

## 3 Procedure

### 3.1 Treatment strategy

**Figure 1:**
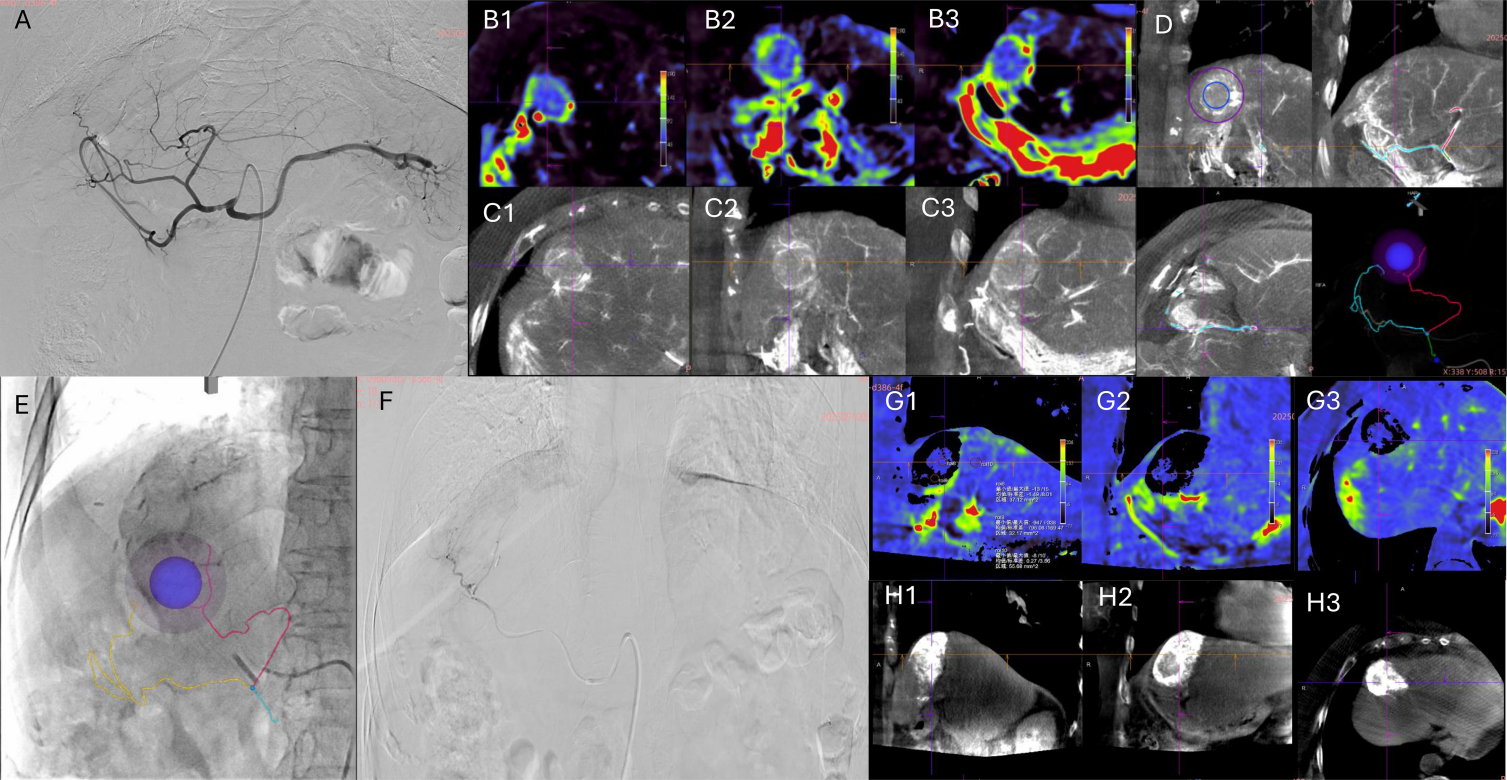
Image-guided TACE procedure. **(A)** Celiac trunk angiography shows no obvious tumor staining; **(B1-B3)** Preoperative perfusion maps (axial, sagittal, coronal) identify the lesion with a characteristic peripheral hyperperfusion signature; **(C1-C3)** Enhanced phase of PBV imaging shows a round-like enhancing shadow corresponding to the lesion; **(D)** Embolization Guidance software was semi-automatically marks the lesion and automatically detects feeder vessels based on enhanced CBCT; **(E)** The marked lesion and feeder vessels are overlaid on live fluoroscopy for superselective guidance; **(F)** Superselective angiography of feeder vessel; **(G1-G3)** Post-operative perfusion map shows residual low perfusion within the “black cavity”; **(H1-H3)** Enhanced phase of PBV imaging confirms a round-like lipiodol deposition area.

Following standard aseptic preparation and local anesthesia, the right femoral artery was punctured using the Seldinger technique, and a vascular sheath was inserted. A RH catheter was selectively placed into the hepatic artery for initial angiography. Pre-procedural PBV acquisition was then performed for perfusion analysis. The enhanced phase of PBV scanning revealed a round-like enhancing shadow, confirming the location of the hypovascular lesion. Using Embolization Guidance software (*syngo* Embolization Guidance; Siemens Healthineers, Forchheim, Germany), the tumor-feeding arteries were automatically identified and overlaid onto real-time fluoroscopy. Subsequently, superselective catheterization of the feeding artery was achieved using a microguidewire and microcatheter under roadmap guidance (as shown in **Figures 1A–F**).

After confirmation, embolization was performed using 8 mL of a lipiodol mixture (containing 10 mL lipiodol and 20 mg lobaplatin) supplemented with embolization microspheres (100–300 μm). Post-embolization angiography confirmed the complete disappearance of tumor vessels and tumor staining. A post-procedural PBV acquisition was conducted for perfusion assessment. The PBV map showed residual low perfusion within a “black cavity”, suggesting incomplete lipiodol deposition and possible portal venous supply. The enhanced phase of PBV also displayed a round-like area of lipiodol deposition (as shown in **Figures 1G, H**). The vascular sheath was removed, the puncture site was closed with a closure device, and a pressure dressing was applied. The patient was then immediately transferred to the CT room for cryoablation therapy.

**Figure 2:**
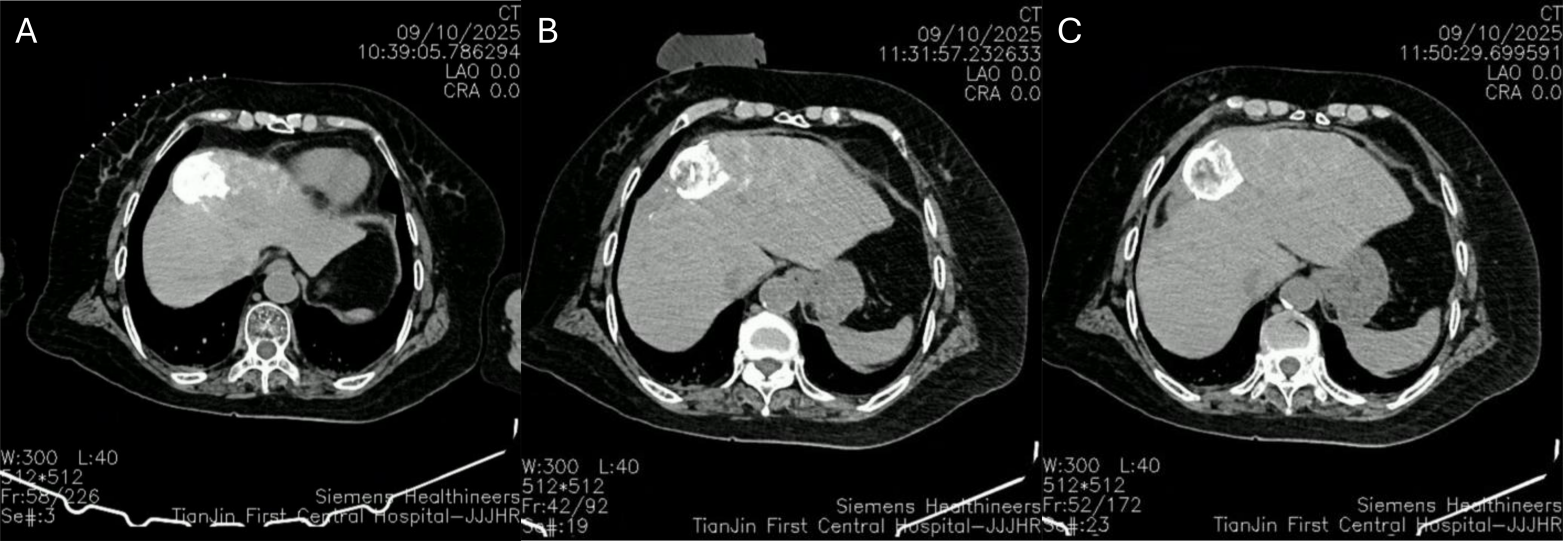
CT-guided cryoablation. **(A)** Preoperative CT shows the targeted embolization area marked by lipiodol; **(B)** Intraoperative CT after needle placement and completion of the first ablation cycle; **(C)** Post-ablation CT scan.

Following local anesthesia for cyroablation, a non-contrast CT scan was performed to localize the intrahepatic nodule and plan the puncture trajectory. Two cryoablation needles (K-13s) were then precisely inserted into the lipiodol deposition area within the liver. Post-puncture CT confirmed optimal needle positioning and morphology. Ablation therapy was conducted using three freeze-thaw cycles (5 min, 8 min, and 8 min each). Post-ablation CT scan showed no evidence of abnormal pneumoperitoneum or hydroperitoneum in the thoracic or abdominal cavities (as shown in **Figure 2**).

### 3.2 PBV acquisition and image guidance

PBV imaging was performed using a flat-panel angiographic system (Artis Pheno; Siemens Healthineers, Forchheim, Germany) before and after TACE. The acquisition protocol involved a 4-second “PBV Body” acquisition with two rotational runs: an initial mask run without contrast, followed by a fill run after the intra-arterial injection of 22 mL diluted contrast media into the proper hepatic artery at 2 mL/s. Acquired images were automatically transferred to a post-processing workstation (syngo X-Workplace; Siemens Healthineers, Forchheim, Germany) for PBV reconstruction, using an validated automatic algorithm (9). Besides the standard mask and PBV map volumes, motion-corrected fill volumes were also reconstructed for enhanced analysis.

## 4 Discussion

To the best of our knowledge, this is the first reported case utilizing combined PBV mapping and embolization guidance software for tumor identification, feeding artery delineation, and intraprocedural guidance in treating hypovascular liver metastases. Previous studies have demonstrated that perfusion imaging can accurately quantify tumor blood perfusion and evaluate TACE response (10–14). For instance, a significant correlation has been shown between baseline blood volume and subsequent perfusion changes post-TACE (10). A study in colorectal liver metastases indicated that higher pre-TACE PBV values correlated with greater tumor shrinkage, provided the post-treatment PBV reduction exceeds 70% (14). Furthermore, residual tumor perfusion (RTP) assessed by dual-phase PBV-CBCT has been identified as a strong predictor of mid-term treatment outcome (13).

The hypovascular nature and potential dual (arterial and portal venous) blood supply of liver metastases often render them invisible on conventional two-dimensional DSA, complicating superselective catheterization. Although earlier literature suggested limited utility of PBV for hypovascular lesions (10, 11), we observed that comparative analysis between fill-phased CBCT and PBV perfusion map can reveal a characteristic “rim-like” enhancement pattern in these tumors: a hypodense region on the fill phase corresponding to peripheral hyperperfusion with central hypoperfusion on the PBV map. This signature enables lesion identification even when conventional DSA fails. The integration of embolization guidance software then allows for automatic feeding artery detection and fluoroscopic overlay, effectively addressing the challenge of superselective embolization for occult lesions, which traditionally requires repeated and time-consuming angiograms. Moreover, the objective change from preoperative hypoperfusion to a postoperative “black cavity” on PBV maps provides a quantifiable endpoint for the embolization procedure. In this case, the peripheral hyperperfused rim raised suspicion for possible residual portal venous supply, prompting the decision for immediate adjunctive cryoablation to consolidate treatment efficacy.

The suboptimal complete necrosis rate and considerable recurrence associated with TACE monotherapy for hepatocellular carcinoma (HCC) are well-recognized, particularly for larger tumor or those with complex vasculature. Conversely, combing TACE with local ablation techniques like cryoablation has emerged as a favorable therapeutic strategy. This combination synergistically enhances local control by first embolizing the arterial supply(reducing the heat-sink effect) and then physically destroying the residual tumor. The feasibility and clinical value of employing CBCT to quantitatively assess changes in PBV before and after TACE, and to subsequently guide complementary MWA for residual tumors, have been demonstrated by studies such as that of Li et al. (15), supporting the concept of a “one-session” combined treatment strategy.

Precise ablation probe placement is critical for success. In the present case, although cryoablation was performed under CT rather than direct DSA guidance, the lipiodol deposited during TACE served as an excellent intrinsic radiopaque marker. It clearly delineated the target’s location and boundaries on the pre-ablation non-contrast CT, facilitating accurate probe positioning and contributing to the successful outcome. This underscores the practical utility of lipiodol as a targeting aid in sequential multimodal procedures.

Compared to the conventional DSA-guided TACE, this integrated workflow offers distinct advantages. First, it overcomes the invisibility of hypovascular tumors on DSA, eliminating the need for repeated angiograms to locate feeder arteries. Second, it provides an objective, quantitative measure (PBV change) to define the embolization endpoint. Third, it creates a visible target (lipiodol deposition) for subsequent ablation, even when performed in a different imaging environment. Furthermore, the combination of TACE and ablation has been validated in literature to induce a more robust immunostimulatory effect, leading to better therapeutic outcomes than TACE alone (16–18). Potential limitations of this new workflow may include a learning curve associated with PBV acquisition and the requirement for specific equipment compatible with both PBV and the embolization guidance software. Our preliminary experience suggested that integrating functional PBV assessments into the treatment algorithm holds promise for further refining patient selection for combined therapies and objectively defining procedural endpoints. This may be particularly valuable for managing hypovascular metastases or recurrent lesions that are challenging to visualize on conventional angiography. Furthermore, the evolving paradigm of combining locoregional therapies (TACE plus ablation) with systemic agents (e.g., tyrosine kinase inhibitors or immune checkpoint inhibitors) may offer synergistic effects, potentially leading to more sustained tumor control and improved survival in advanced malignancies.

## 5 Conclusion

In conclusion, CBCT-derived PBV mapping combined with embolization guidance technology effectively aids in locating hypovascular liver metastases and identifying their feeding arteries, thereby facilitating precise superselective catheterization. Furthermore, quantifiable changes in PBV values before and after TACE assist operators in objectively determining the procedural endpoint and optimizing treatment strategy. Finally, post-TACE lipiodol deposition serves as a reliable radiopaque marker, confirming the target lesion and verifying ablation needle placement prior to immediate complementary ablation, ultimately optimizing local treatment efficacy. Further studies with larger patient cohorts are warranted to validate these finding and refine this integrated approach.

## Data Availability

The original contributions presented in the study are included in the article/supplementary material. Further inquiries can be directed to the corresponding author.
